# Assessment of oral health status among salt factory workers in Nagaur, Rajasthan: A cross sectional study

**DOI:** 10.6026/973206300220112

**Published:** 2026-01-31

**Authors:** Shadab Ali, Mohsin Khan, Minal Chauhan, Javed Khan, Ankita Singh, Dharmveer Chouhan, Shahid Ali, Shazia Sahir

**Affiliations:** 1 Department of Public Health Dentistry, Vyas Dental College and Hospital, Jodhpur, Rajasthan, India; 2 General Dental Practitioner, Shine dental clinic, Nagaur, Rajasthan, India

**Keywords:** Salt pan, oral hygiene, mucosal lesions, WHO oral health assessment, DMFT score

## Abstract

The oral health status of salt factory workers is an underexplored yet significant aspect of occupational health concerns. Hence, a cross-sectional survey was carried out among salt factory workers. More than 5 years of work experience had higher mean DMFT scores and prevalence for oral mucosal lesions (p <0.001). Dental erosion was found significantly higher in more experience group (65.9% in compare to 44% with p value <0.001), highlighting the need for targeted oral health awareness programs and improved access to preventive dental care for these workers.

## Background:

The World Health Organization defines health as a holistic state of complete physical, mental and social well-being, rather than simply the absence of disease or infirmity [1]. The health of an individual is profoundly influenced by their environment, which encompasses the conditions, influences and surroundings that affect an organism's life. Environmental factors play a critical role in determining the health status of populations. According to WHO (2015) [2], the environment includes all external factors that impact an organism, while Last (2001) [3]. It further elaborates by describing the environment as "everything external to the human host." Specifically, the occupational environment encompasses the totality of external conditions present in the workplace that can influence the health and well-being of workers. Workplace conditions play a crucial role in influencing the health and well-being of workers. One such setting with considerable health implications is the salt pan-a shallow, undrained basin where water collects, evaporates and leaves behind salt deposits [4]. Salt pans are integral to the salt production industry, which is a major industry in India. India is the third-largest salt producer in the world, after China and the USA, with a record production of about 391.13 lakh tons (39.113 million metric tons) in the fiscal year 2022-23-the highest ever in the country. Gujarat contributes over 75% of this production, followed by Tamil Nadu at around 13% [5] and Rajasthan at 11%. In Rajasthan alone, approximately 20,000 workers are employed in the salt industry [6]. Work-related health conditions such as skin allergies, eye irritation and infections are common in these workers and are very well reported [7]. While oral health status of different industry workers such as battery factory workers [8, 9], construction workers [10, 11] and garment industry workers [12]. Therefore, it is of interest to evaluate the oral health status of these workers, examine their oral hygiene practices, assess their need for dental treatment and utilization of dental services and compare their oral health status based on the duration of their work experience in the salt industry.

## Materials and Methods:

The study was conducted among salt factory workers located in Nawa, Nagaur district of Rajasthan. Nawa, renowned for its extensive salt pans, which contribute significantly to Rajasthan's salt production, is a town located in the arid region of western India, near to the Sambhar Lake, India's largest inland salt water lake and characterized by a semi-desert climate with extreme temperatures and low rainfall. More than 1500 workers get employment in Nawa salt factories, they work under similar environmental condition and most of them are paid on a daily basis. Ethical permission was taken from Institutional Ethical Committee of Vyas Dental College and Hospital, Jodhpur, also permission for organizing oral health check-up camps for factory workers taken from BCMO, Nawa. Salt factory workers aged 18 years and above who had been employed in the salt industry for more than one year were included in the study. Workers with a history of systemic illnesses such as diabetes mellitus or hypertension, which could influence the study outcomes, were excluded. A pilot study involving 35 salt factory workers was conducted to assess the feasibility and practicality of the study, as well as to estimate the time required for examining each participant. On average, completing one assessment form took about 8-10 minutes. Data for the main study were collected through a cross-sectional survey using a survey proforma developed based on the WHO Oral Health Assessment Form (2013). A total of 384 workers took part in this study and 10 oral health check-up camps at community centre in Nawa were organized, in the span of two months for data collection to meet the desired sample size. The DMFT (Decayed, Missing, Filled Teeth) index was used to assess and monitor dental caries experience in populations. The periodontal health of population was measured by CPI index; for bleeding, pocket depth and loss of attachment components. As Nagaur district, being part of the Fluoride belt in India and known for the endemicity of fluorosis, Dean's Fluorosis Index was also measured to know the extent of fluorosis among workers. Assessment of oral mucosa was done as per WHO proforma for any lesion or condition and their location. Following the oral examinations, a brief oral health education session was delivered to all the workers in their local language. The survey findings were shared with them at the examination site, and individuals identified as needing further dental treatment were referred to appropriate dental care facilities.

## Statistical analysis:

The collected data were compiled in Microsoft Excel and analyzed using SPSS version 22. Descriptive statistics such as mean, median, standard deviation, and proportions (% of subjects affected) were computed for each clinical parameter. Inferential analysis was carried out using Pearson's Chi-square test (χ^2^), unpaired t-test, One-Way Analysis of Variance (ANOVA), and Stepwise Multiple Linear Regression. A 95% confidence level was used, and a p-value ≤ 0.05 was considered statistically significant.

## Results:

**Figure 1:**
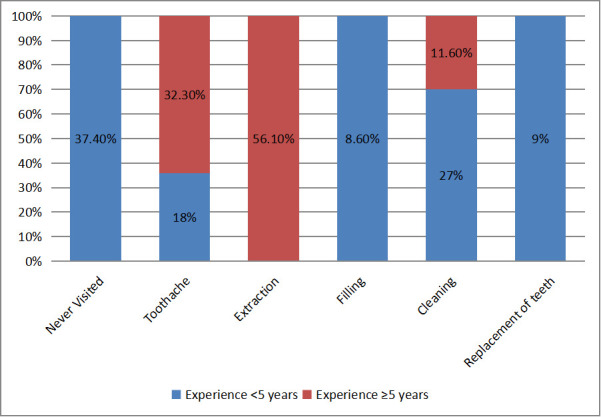
Distribution of study population based on the reason for past dental visit

**Figure 2:**
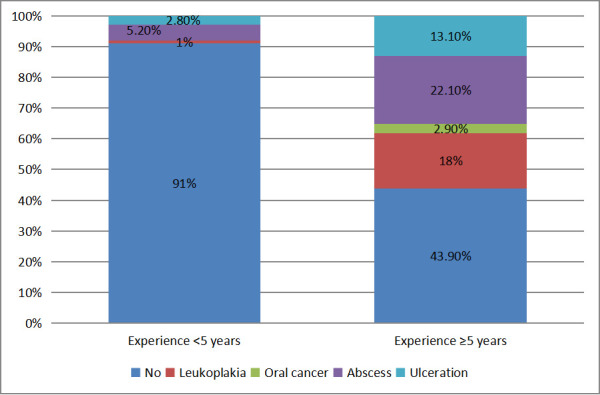
Distribution of study population based on presence of oral mucosal condition

**Figure 3:**
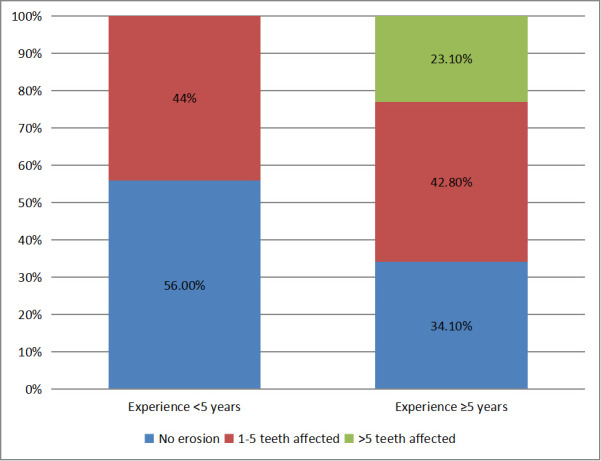
Distribution of study population based on Dental Erosion

Workers with less than five years of experience had a mean DMFT score of 5.06 ± 3.40, while those with five or more years of experience had a mean score of 5.39 ± 3.01. Statistical analysis indicated no significant difference between the two groups (t = -0.998, p = 0.319, NS). Although the mean DMFT score was marginally higher among the more experienced workers, the difference was not statistically meaningful (Table 1). Distribution of the study population based on the reason for past dental visits is presented in Figure 1. Among participants with less than five years of experience, 37.4% had never visited a dentist. Among those who had, the most common reasons were dental cleaning (27%), toothache (18%) and replacement of teeth (9%), while 8.5% had visited for dental fillings. In contrast, among participants with five or more years of experience, The most frequently reported reason for dental visits was tooth extraction (56.1%), followed by treatment for toothache (32.4%) and dental cleaning (11.6%). A chi-square test revealed a highly significant association between the reason for previous dental visits and the workers' level of work experience (χ^2^ = 232.745, p < 0.001) (Figure 1). Periodontal health of factory workers was examined by CPI Index. Both pocket depth and loss of attachment component were assessed. Among individuals with less than five years of experience, 9% participants had 4-5 mm pockets and 8.5% had pocket depth ≥6 mm. In contrast, among those with five or more years of experience 22% exhibited 4-5 mm pockets, rest had no pocket formation. A statistically significant difference was observed between the two groups (χ^2^ = 25.747, p < 0.001) (Table 2). While CPI- Loss of Attachment score showed that among the participants with less than five years of experience, 82.5% had no significant loss of attachment, 9% had 6-8 mm attachment loss and 8.5% exhibited 9-11 mm attachment loss In contrast, in the group with five or more years of experience, 78% had 0-3 mm loss of attachment, while 22% exhibited 6-8 mm attachment loss, with no cases of 9 mm or greater attachment loss. The observed difference was statistically significant (χ^2^ = 24.474, p < 0.001) (Table 3). Among participants with less than five years of experience, 91% had no oral mucosal lesions, while 9% had lesions present. For those with five or more years of experience, 43.9% had no lesions, whereas 56.1% had oral mucosal lesions. The chi-square test showed a highly statistically significant difference (χ^2^ = 99.875, p < 0.001) (Figure 2). Figure 3 shows the prevalence of dental erosion among participants. Total 65.9% of participants from more experienced group had dental erosion while only 44% participants of less experienced group had dental erosion (Figure 3).

## Discussion:

Salt factory workers face socioeconomic challenges due to low wages, job insecurity and inadequate healthcare access. All these factors lead many workers to neglect basic health needs, including dental care and limited awareness and affordability prevent them from seeking regular dental checkups, resulting in unmanaged oral conditions like dental caries and periodontal disease, which can eventually cause tooth loss. In our study, the mean DMFT score for participants with less than 5 years of experience was 5.06 ± 3.40, while for those with more than 5 years of experience it was 5.39 ± 3.01. The non-significant p-value of 0.139 indicates that dental health deterioration, as reflected by DMFT scores, was not significantly different between the two experience groups, although the scores were slightly higher in the more experienced group. This finding suggests that other factors-such as oral hygiene practices, dietary habits and preventive care-may play a more influential role in DMFT progression than years of experience alone. These results are consistent with the findings of Chavan *et al.* (2016), who reported a mean DMFT of 5.04 ± 3.29 [13]. Present study shows that 32.4% workers from more experience group visited dental clinic due to complaint of toothache and 56.1% workers from same group have visited dental clinic previously for the extraction of one or more teeth. This make 25.2% of total participating factory workers have undergone tooth extraction. This finding aligns with the study conducted by Amin *et al.* (2001) on workers exposed to acid fumes in phosphate and battery industries in Jordan, where 12% of the workers reported visiting a dentist primarily for tooth extraction. This suggests that limited dental visits are often driven by the need for relief from pain or removal of severely damaged teeth, rather than for preventive or routine care [14]. Our study found that 100% of participants in both experience groups exhibited bleeding on probing. These results suggest that gingival bleeding is a prevalent issue among all participants, irrespective of work experience. The widespread occurrence of bleeding may be linked to poor oral hygiene, ineffective plaque control and insufficient professional periodontal care. Since bleeding on probing serves as an early sign of gingival inflammation and periodontal disease, these results emphasize the importance of enhancing oral hygiene practices and ensuring regular periodontal care in the study population. A comparable pattern was noted by Kumar *et al.* (2009) [15] in their study on the rural population of Ambala District, Haryana, which reported a high overall prevalence of periodontal disease (92.7%). These findings suggest that poor oral hygiene, coupled with lower socioeconomic status and challenging working conditions, significantly contributes to the prevalence of periodontal diseases.

Among participants with less than five years of experience, 82.5% showed no periodontal pockets, 9% had shallow pockets (approximately 4-5 mm) and 8.5% had deeper pockets (6 mm or more). In comparison, in those with five or more years of experience, 78% had no pockets, while 22% exhibited shallow pockets. This variation between the two groups was statistically significant. In the present study, 82.5% of participants with less than five years of work experience showed a loss of attachment between 0-3 mm, 9% had attachment loss of 6-8 mm and 8.5% demonstrated loss of 9-11 mm. These findings indicate that attachment loss tends to increase with longer duration of employment, which could be linked to prolonged exposure to risk factors such as tobacco use, poor oral hygiene habits and occupational stress. This underlines the need for periodic periodontal assessments and preventive interventions to reduce attachment loss and maintain oral health. Comparable findings were reported by Sanadhya *et al.* (2013) [16], who observed a CPI index prevalence of 96.4% among salt workers at Sambhar Lake, Jaipur.

Baishya *et al.* (2019) [17] documented a 86.7% CPI index prevalence among brick klin workers in Odisha. Studies by Dagli *et al.* (2008) [18] and Kumar *et al.* [19] also recorded a high prevalence of periodontal disease (98.25%) among green marble mine workers in Rajasthan. Present research also shows that, 91% of the participants with less than five years of experience had no oral mucosal conditions, while 9% had some form of oral mucosal condition. The most common conditions observed were abscess (5.2%) and ulceration (2.8%), with leukoplakia reported in only 1% of individuals. Among individuals with five or more years of work experience, the occurrence of oral mucosal conditions was markedly higher. Only 43.9% were free of any lesions, whereas 19% presented with leukoplakia, 2.9% had oral cancer, 24.3% showed abscesses and 14.7% exhibited ulcerations. In comparison, a study by Kumar *et al.* (2024) [20] conducted among postal workers in Bhubaneswar, Odisha reported an overall prevalence of oromucosal lesions at 7.8%, with leukoplakia accounting for 7% of the cases. The variation in the prevalence of oral precancerous and cancerous lesions can be attributed to differences in demographic factors, workplace environment and the intensity of labor involved. The demographic aspects such as age, socioeconomic status and education level influence tobacco and alcohol consumption habits, which are major risk factors for these lesions. Additionally, the nature of the working environment plays a crucial role, as individuals in physically demanding jobs may experience higher stress levels, leading to increased tobacco or betel quid use as a coping mechanism. Limited awareness and access to preventive dental care further contribute to the higher prevalence of these lesions. The most affected site in the ≥5 years group was the buccal mucosa (22.5%), followed by the tongue (17.3%) and palate (13.9%). In contrast, the <5 years group had much lower lesion occurrences, with buccal mucosa (5.7%), tongue (2.8%) and palate (0.5%) involvement. These findings suggest a strong correlation between prolonged occupational exposure and the development of mucosal lesions, possibly due to chronic irritation from tobacco, alcohol, or mechanical trauma. The high prevalence of buccal mucosa lesions could be associated with tobacco chewing habits, while tongue and palatal lesions may indicate smoking-related changes, alcohol-induced irritation, or prolonged mechanical trauma from ill-fitting dentures or sharp teeth. Considering the statistically significant difference (p < 0.001), these findings highlight the importance of implementing early screening measures, tobacco cessation initiatives and occupational health interventions to curb the progression of mucosal lesions into more serious conditions. Our findings have one important clinical outcome that among <5-year participants, prevalence of dental erosion is 65.9% in compared to 44.1% in less experience group (p value < 0.001). Same relation between dental erosion and work experience was drawn by Fulova *et al.* (2024) [21] among employees of chemical and pharmaceutical production in Russia. They found that severity of erosion increased with the length of service. Khurana *et al.* (2014) [22] observed in their study on an industrial workforce that poor oral health was common among the participants and showed a significant association with dental erosion. Salt factory workers experience dental erosion due to prolonged exposure to airborne salt particles, which lower saliva pH and promote enamel demineralization. The high-salt environment also reduces salivary flow, weakening its protective and buffering effects. Additionally, exposure to acidic fumes from chemicals like hydrochloric or sulfuric acid further accelerates enamel erosion [23]. The dry workplace conditions contribute to dehydration, exacerbating oral acidity. Over time, these factors lead to irreversible enamel loss, increased tooth sensitivity and higher susceptibility to dental tissues loss [24, 25]. Implementing preventive strategies- such as maintaining proper hydration, using fluoride-based therapies and attending routine dental examinations-can help lower the risk of dental erosion among individuals working in salt factories.

## Conclusion:

We show notable variations in oral health status, lifestyle habits, and access to dental care between workers with fewer than five years of experience and those who had been employed for five years or longer. While both groups exhibited poor oral hygiene practices, the longer experienced group had higher rates of periodontal disease, dental erosion, and oral mucosal lesions. These outcomes highlight the vulnerability of salt factory workers and need of evidence-based policies to protect the oral health of this workforce.

## Figures and Tables

**Table 1:** Distribution of study population based on mean DMFT scores

**Mean DMFT**	**Experience < 5 years**		**Experience ≥ 5 years**		**t value, p value**
	Mean	SD	Mean	SD	
	5.06	3.4	5.39	3.01	-0.998, 0.319 NS
*Statistically Significant,
** statistically highly significant,
NS-Not Significant

**Table 2:** Distribution of study population based on CPI scores for pocket

**CPI score**		**Experience < 5 years**		**Experience ≥ 5 years**	
		**n**	**%**	**n**	**%**
Pocket	Absent	174	82.50%	135	78%
	1= 4-5 mm pocket	19	9%	38	22%
	2= ≥6 mm pocket	18	8.50%	0	0%
Total		211	100%	173	100%
Chi square value, p value		25.747, <0.001 **			
Statistically Significant,
** Statistically highly significant,
NS-Not Significant

**Table 3:** Distribution of study population based on CPI-LOA scores

**CPI-LOA score**	**Experience < 5 years**		**Experience ≥ 5 years**	
	**n**	**%**	**n**	**%**
0-3 mm	174	82.50%	135	78%
4-5 mm	0	0%	0	0%
6-8 mm	19	9%	38	22%
9-11 mm	18	8.50%	0	0%
12 mm or more	0	0%	0	0%
Total	211	100%	173	100%
Chi square value, p value	24.474, <0.001 **			
Statistically Significant,
** Statistically highly significant,
NS-Not Significant
